# DFI: gene feature discovery in RNA-seq experiments from multiple sources

**DOI:** 10.1186/1471-2164-13-S8-S11

**Published:** 2012-12-17

**Authors:** Hatice Gulcin Ozer, Jeffrey D Parvin, Kun Huang

**Affiliations:** 1 The Department of Biomedical Informatics and The Ohio State University Comprehensive Cancer Center, The Ohio State University, Columbus, OH, 43210, USA

## Abstract

**Background:**

Differential expression detection for RNA-seq experiments is often biased by normalization algorithms due to their sensitivity to parametric assumptions on the gene count distributions, extreme values of gene expression, gene length and total number of sequence reads.

**Results:**

To overcome limitations of current methodologies, we developed Differential Feature Index (DFI), a non-parametric method for characterizing distinctive gene features across any number of diverse RNA-seq experiments without inter-sample normalization. Validated with qRT-PCR datasets, DFI accurately detected differentially expressed genes regardless of expression levels and consistent with tissue selective expression. Accuracy of DFI was very similar to the currently accepted methods: EdgeR, DESeq and Cuffdiff.

**Conclusions:**

In this study, we demonstrated that DFI can efficiently handle multiple groups of data simultaneously, and identify differential gene features for RNA-Seq experiments from different laboratories, tissue types, and cell origins, and is robust to extreme values of gene expression, size of the datasets and gene length.

## Background

High-throughput RNA-sequencing (RNA-seq) enables researchers to quantify genome-wide gene expression with high resolution [1]. At the same time, it raises many new challenges for data processing and analysis. One major challenge is how to effectively combine, compare and contrast samples to identify differential gene features. The common sense answer to this question is to apply an effective inter-sample normalization procedure before starting any type of comparative analysis on the samples from different sites, as well as on the samples from the same dataset [2-4]. On the other hand, it has been shown that the choice of normalization method itself could be a major factor that determines estimates of differential expression [5].

After the alignment of high throughput short sequence reads to the reference genome, expression levels can be quantified in terms of total number of reads that are aligned to the genes. Then, generally, a proper normalization algorithm is used to estimate expression levels for comparative analyses. One of the problems with high throughput sequencing is longer genes are sequenced more and have larger gene counts [6]. The first and most commonly used normalization method RPKM (reads per kilobase of exon per million mapped reads) [7] addresses this bias by simply scaling counts by the gene length. Later studies have shown that more sophisticated weighting methods are needed to lessen this bias [5,8]. Another challenge with sequencing is modelling the distribution of the gene counts, as differences in relative distributions of the samples would affect the detection of differential expression [3]. Poisson [1] and negative binomial distributions [9,10] are the most commonly used ones to model the gene count data. These models are parametric i.e. require assumptions on the distribution of the data. However, in the real scenario, these distribution assumptions might not always hold true [5] and estimation of the model parameters can be very difficult [11].

Here, we introduce Differential Feature Index (DFI) to identify distinctive features across a large set of diverse experiments using read counts without any direct inter-sample normalization. The DFI method is non-parametric (i.e. calculations of DFI do not require any assumptions on the distribution of the data) and unsupervised (i.e. does not require group information to identify differential features). In this study, first, we compared DFI to currently accepted methods [4] such as EdgeR [9], DESeq [10] and Cuffdiff [12], as well as the classical t-test. Then, we evaluated the efficiency of DFI in comparing multiple groups of data from different research groups at the same time. We found that DFI was effective and robust for selecting differential gene features for RNA-Seq experiments from different laboratories, tissue types, and cell origins.

## Results

### Differential Feature Index (DFI) approach

**Figure 1:**
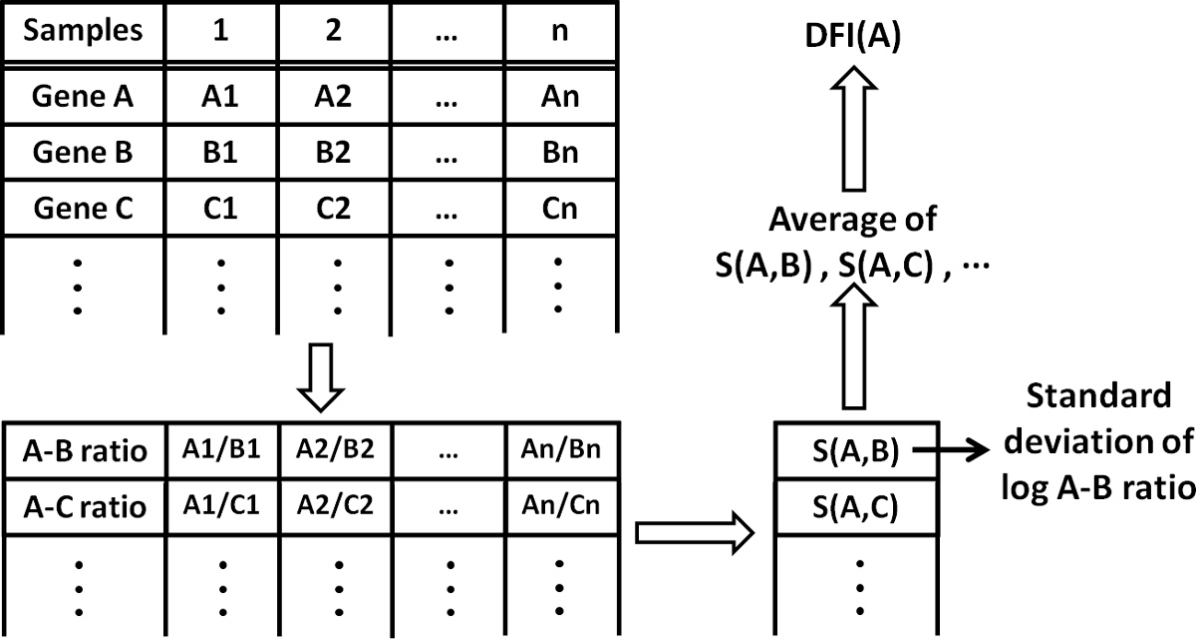
**The DFI calculation workflow**. Rather than transforming whole datasets by normalization, each data point is compared to the other data points in the same dataset in a pair-wise fashion. The standard deviation of this ratio becomes a measure of the variability of a given gene among the multiple datasets being compared.

DFI can identify distinctive gene features across a large set of diverse experiments without any direct inter-sample normalization. DFI is defined as the average pair-wise variation between any particular gene and all the other genes. Workflow for DFI calculation is shown in Figure 1. The DFI is a non-parametric (i.e., calculations of DFI do not require any assumptions on the distribution of the data) and unsupervised (i.e., does not require group information to identify differential features) approach to identify differential features.

A large DFI implies that the gene varies substantially across all experiments and can be considered as a feature to differentiate them, while a small DFI means expression of this gene is quite stable across all experiments. Thus, one can order the gene features based on DFI values and identify differential features. In this paper, we selected top one percent of the gene features as differentially expressed.

### Accuracy of DFI compared to other methods when evaluating results pair-wise

To evaluate accuracy of DFI method in identifying differentially expressed genes, we used 42 RNA-seq experiments [5] conducted on two biological samples from Microarray Quality Control Project (MAQC): Ambion's human brain reference RNA (14 experiments) and Stratagene's universal human reference (UHR, 28 experiments) (SRA Project ID: SRP001847) (Table 1). We first computed DFI values using the gene counts from these samples. As part of the MAQC project, about one thousand genes were assayed by qRT-PCR for relative quantification of these two samples [13]. We considered this qRT-PCR dataset as a gold-standard and explored the relationship between true differential expression and DFI values. We used the same thresholds with a previously published method [5] and considered genes with log2-fold changes greater than 2 and less than 0.2 as differentially expressed (true positives) and non-differentially expressed (true negatives) respectively.

**Table 1:** mRNA-seq experiments from NCBI SRA.

Tissue/cell line	Number of experiments	SRA project ID	Project name
Kidney	3	SRP000225	Illumina sequencing human kidney RNA samples to study mRNA expression levels
		
Liver	3		

GM12878 (Blood)	7	SRP000228	RNASeq expression profiling for ENCODE project
		
Hep2G (Liver Carcinoma)	4		
		
K562 (Leukemia)	9		

Brain	1	SRP000626	Deep surveying of alternative splicing complexity in the human transcriptome by high-throughput sequencing
		
Cerebral Cortex	1		
		
Heart	1		
		
Liver	1		
		
Lung	1		
		
Skeletal Muscle	1		

Liver female 1	6	SRP001558	Sex-specific and lineage-specific alternative splicing in primates
		
Liver male 1	6		

Brain MAQC2	14	SRP001847	Evaluation of Statistical Methods for Normalization and Differential Expression in mRNA-Seq Experiments
		
UHR MAQC2	28		

**Figure 2:**
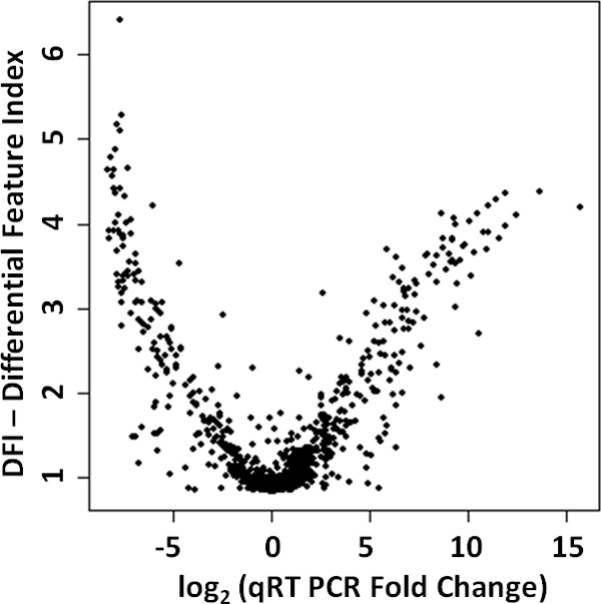
**Scatter plot showing log2 qRT-PCR fold changes for 935 genes (x-axis) and DFI values (y-axis)**.

**Figure 3:**
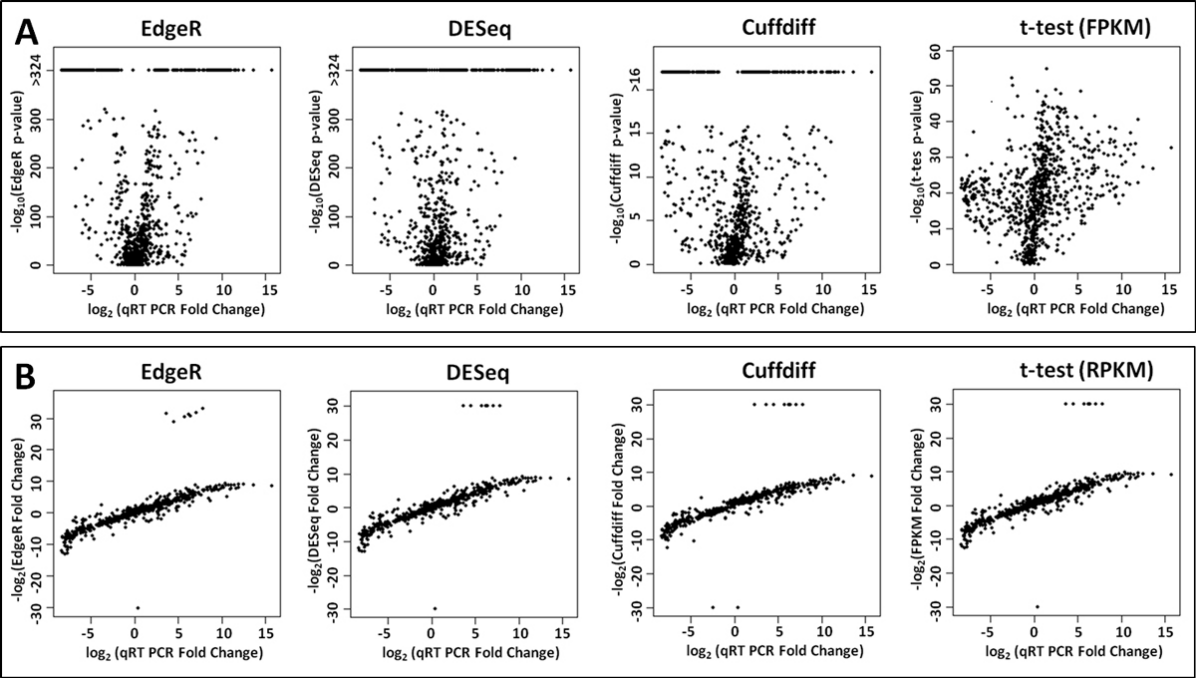
(A) Volcano plots showing log2 qRT-PCR fold changes for 935 genes (x-axis) and -log10 p-values for EdgeR, DESeq, Cuffdiff, and t-test (y-axis). (B) Scatter plots showing log2 qRT-PCR fold changes for 935 genes (x-axis) and log2 fold changes reported by EdgeR, DESeq, Cuffdiff and t-test (y-axis).

DFI is a single measure to rank differentially expressed features. When we examined relationship between DFI and qRT-PCR fold changes, we observed that changes in qRT-PCR are directly correlated with DFI values (Figure 2). Then, we examined the relationship between FDR adjusted p-values and fold changes reported by EdgeR, DESeq, Cuffdiff and t -test, and qRT-PCR fold changes. P-values did not show any correlation with qRT-PCR fold changes (Figure 3A). P-values only denote the probability of observing the reported fold-change by chance. On the other hand, fold changes reported by these methods are highly correlated with qRT-PCR fold changes (Figure 3B).

**Figure 4:**
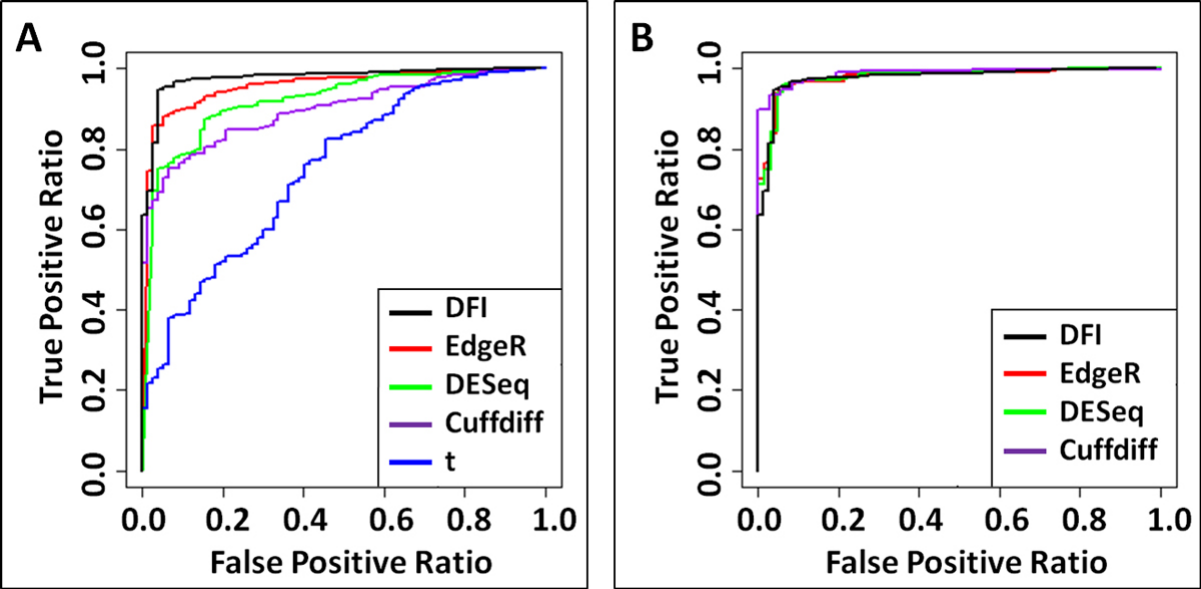
**ROC curves are plotted for DFI (black), EdgeR (red), DESeq (green), Cuffdiff (purple), and t-test (blue) methods based on qRT-PCR validated genes**. (A) Comparison of DFI and p-values reported by EdgeR, DESeq, Cuffdiff and t-test shows that DFI is more accurate than p-values reported by the others. (B) Comparison of DFI and fold changes with p < 0.01 reported by EdgeR, DESeq and Cuffdiff shows that DFI is comparable to the combination of fold change and p-value criteria of the others. Since fold changes for Cuffdiff and t-test were both RPKM based they resulted in same values. Therefore, t-test is not plotted. Also, genes with less than 10 reads in both samples are not considered in this evaluation.

Next, we employed qRT-PCR data to construct receiver-operator characteristic (ROC) curves. These plots illustrate performance of the differential expression methods on classifying true differential expression against false discoveries. For EdgeR, DESeq, Cuffdiff and t-test, first, we plotted ROC curves based on p-values alone. The ROC curves illustrate that the DFI ranking method is more accurate than the p-values reported by the other methods under consideration (Figure 4A). Next we plotted ROC curves based on fold changes reported by these methods. We only considered the genes with a p-value smaller than 0.01 -the most commonly used threshold for these methods. ROC curves demonstrate that all methods perform similarly (Figure 4B). When the entire fold changes reported by EdgeR, DESeq, Cuffdiff and t-test are examined (Figure 3B), indeed, their clear correlation with qRT-PCR fold changes would necessitate this level of accuracy. Nevertheless, DFI ranking is superior, since it is a single criteria and as accurate as the combination of fold change and p-value criteria of the other methods.

### Simultaneous comparison of two groups from multiple studies

**Figure 5:**
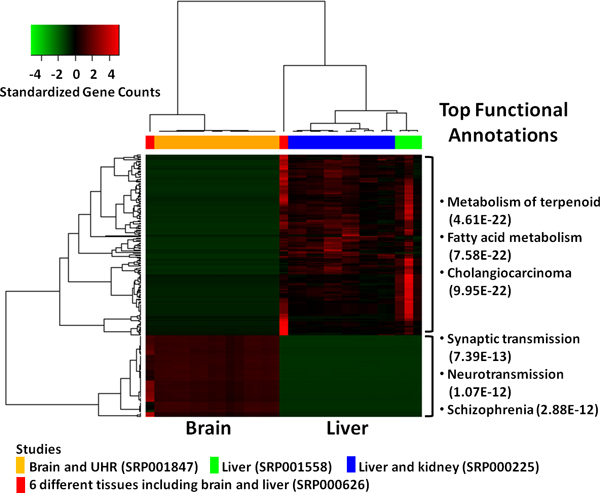
**Clustering of 15 brain and 16 liver RNA-seq experiments from 4 different studies**. Heatmap shows hierarchical clustering of the samples and top one percent of the gene features based on Pearson correlation coefficient (distance function for hierarchical clustering is 1-correlation). Brain and liver samples are clearly separated and functional annotations confirm the accuracy of the selected gene features.

We further tested DFI method in comparing RNA-seq data for two tissues with 15 experiments on brain and 16 on liver collected from four different projects in NCBI's SRA (one brain study, two liver studies, one study containing both brain and liver tissues) (Table 1). We selected top one percent of the genes (when ranked by DFI) as differential gene features (Additional file 1). When selected genes and samples were hierarchically clustered by Pearson correlation coefficient (Figure 5), these genes are separated into two groups. Gene function enrichment analysis using Ingenuity Pathway Analysis (IPA) confirmed that the genes with high expression levels in each tissue are highly tissue specific (Additional file 1).

### Simultaneous comparison of multiple groups form same study

**Figure 6:**
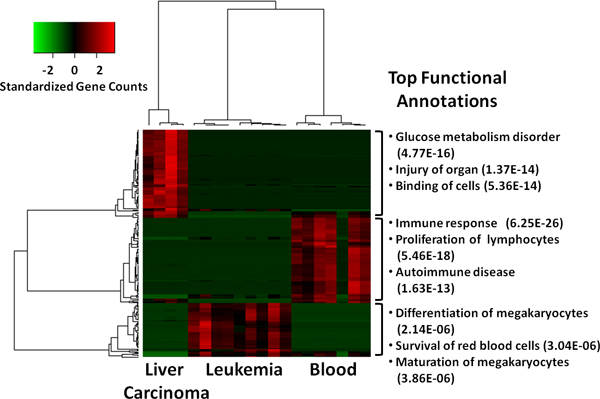
**Clustering of 4 liver carcinoma, 9 leukemia and 7 blood RNA-seq experiments from ENCODE project**. Heatmap shows hierarchical clustering of the samples and top one percent of the gene features based on Pearson correlation coefficient (distance function for hierarchical clustering is 1-correlation). Three samples are clearly separated and functional annotations confirm the accuracy of the selected gene features.

Next, we tested the DFI method in selecting differential gene features of RNA-seq data for three cell lines; 9 experiments on leukemia, 4 experiments on liver carcinoma and 7 experiments on blood (SRA Project ID: SRP000228) (Table 1). We selected top one percent of the genes (when ranked by DFI) as differential gene features (Additional file 2). Hierarchical clustering of the experiments and genes based on these selected gene features (Figure 6) shows clear separation of the three cell lines. Also, top functional annotations reported by IPA for the genes with high levels in each cell line are highly consistent with the cell types (Additional file 2).

### Simultaneous comparison of multiple groups from multiple studies

**Figure 7:**
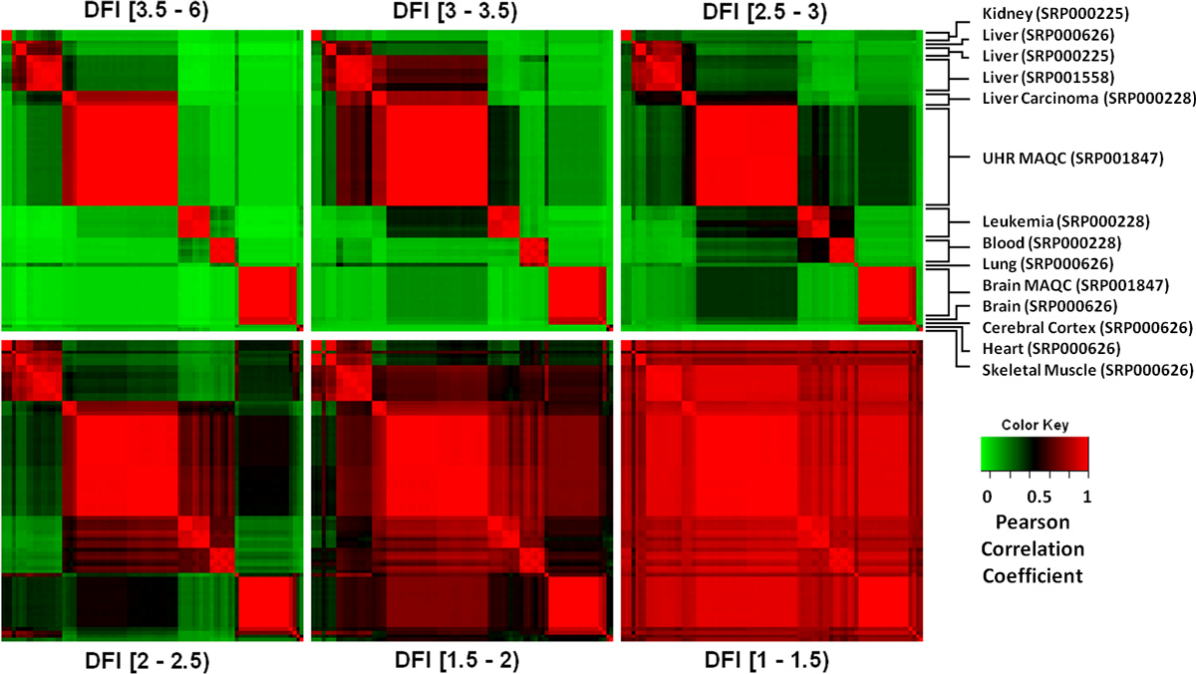
**Clustering of 86 RNA-seq experiments from 5 different studies with 11 different sample types**. Heatmaps show hierarchical clustering of the samples based on Pearson correlation of the genes with sliding DFI ranges. When top gene features (with DFI value larger than 3.5) are used, sample type drives the clustering (top-left). As the DFI values get smaller, correlation of the samples get weaker. When genes with DFI values less than 1.5 are considered all of the samples correlate to each other. Series of these heatmaps confirms the differentiation power of the DFI ranking in a single step.

Finally, we calculated DFI values for 86 RNA-seq experiments from 5 different projects in NCBI's SRA including experiments on 15 brain, 16 liver, 3 kidney, 1 lung, 1 heart, 1 skeletal muscle, 1 cerebral cortex, 7 GM12878, 4 Hep2G, 9 K562, and 28 universal human reference (UHR) samples (Table 1). When genes with large DFI are selected and samples were hierarchically clustered by Pearson correlation coefficient, tissue origin drove the clustering (Figure 7). As examples, liver samples from 3 different studies, brain samples from 2 different studies and a cerebral cortex sample clustered tightly together in appropriate groupings. Even a single lung sample stood out by itself and did not correlate with others. As the DFI values diminished, all of the samples correlated to each other (Figure 7). This confirmed the differentiation power of the DFI ranking in a single step.

## Discussion

We demonstrated that DFI is a highly effective and robust method for selecting gene features for RNA-seq experiments from multiple groups of samples. First, DFI is robust to the variation in total number of sequence reads across experiments. A recent study [3] suggested that incorporation of total number of sequence reads in normalization may impact comparative results. Furthermore, another study [11] has shown that sequencing depth itself affects the identification of genes as differentially expressed. DFI formulation is independent of total number of sequence reads, since calculations starts with pair-wise ratios of the gene counts within each experiment (Figure 1). This approach circumvents the need to normalize total number of reads. Out of 4.4 million to 54.8 million total number of sequence reads in above 86 RNA-seq experiments 0.3 million to 16.3 million reads were aligned to the reference genome (Additional file 3: Figure S1). This large variation did not affect the DFI calculations and the clustering of the experiments. For example, 16 liver experiments from 3 different studies clustered very tightly (Figure 5) although total number of sequences reads in the samples varied between 2.8 million and 14 million.

Second, DFI is robust to low and high gene counts. Two main problems occur with normalization methods and differential expression statistics [5]: genes with high counts are more likely to be discovered as differentially expressed and genes with low counts tend to affect differential expression statistics. To test the robustness of DFI, EdgeR and DESeq to extreme values of gene counts, we eliminated genes with high or low expression and compared differential expression rankings to the full gene set. We demonstrated that DFI is the least affected by extreme values of gene counts compared to EdgeR and DESeq (Additional file 3: Figure S2).

Third, DFI is robust even when the number of samples is small. In 100 tests, we randomly selected three samples from each of the two groups in MAQC dataset and compared the DFI ranking with the gold standard for each test. ROC curves demonstrated that when testing low numbers of datasets, the accuracy of the DFI ranking only changed slightly between these 100 tests with the area under ROC curve of 0.952 +/- 0.004 (Additional file 3: Figure S3).

Finally, DFI is independent of gene length. A known issue with the sequencing platforms is that longer genes are sequenced more and end up with larger gene count numbers [6]. Simply scaling gene counts by transcript length, as in RPKM [7], is insufficient to cure this bias. More sophisticated weighting methods are needed to mitigate this bias [5,8]. On the other hand, DFI calculations are completely independent of gene length due to its formulation (Equation 1). Including a scaling factor on gene counts based on gene length only adds a constant term to the standard deviation calculation. For this analysis, every gene count *g_j _*is adjusted to its length by multiplying *l_j_*:

(1) $$\begin{array}{ccc} G_{jk} & =\left\{log_{2}\left(\frac{g_{j1}l_{j}}{g_{k1}l_{k}}\right),log_{2}\left(\frac{g_{j2}l_{j}}{g_{k2}l_{k}}\right),\cdots \;,log_{2}\left(\frac{g_{jm}l_{j}}{g_{km}l_{k}}\right)\right\} & \\ & ={\left\{log_{2}\left(\frac{g_{ji}}{g_{ki}}\right)+log_{2}\left(\frac{l_{j}}{l_{k}}\right)\right\}}_{i=1\to m} \end{array}$$

This adjustment will only add a constant term to the elements of array *G_jk_*. Although the mean of the array will be shifted, the standard deviation calculation will not be affected from this adjustment. Therefore, DFI calculation is theoretically independent of the gene lengths. Also, no association was observed between gene lengths and DFI values calculated in this study (Additional file 3: Figure S4).

It is conceivable that application of DFI can be extended to a wide spectrum of high throughput data. In fact, a similar metric had been applied to select normalization factor in qRT-PCR experiments [14] and to compare different ChIP-seq datasets [15]. Further investigation on DFI features may lead to effective methods for integrating datasets from multiple modalities (e.g., microarray and RNA-seq).

In summary, we have developed a Differential Feature Index that allows one to accurately and effectively identify the genes that change expression in multiple RNA-seq datasets. This index obviates the need to normalize samples and can accommodate any number of datasets with multiple sizes.

## Methods

### RNA-seq datasets

This article considers 86 mRNA-seq experiments from 5 different projects in NCBI Sequence Read Archive (SRA) (Table 1): 1) 42 RNA-seq experiments from a study that evaluates the effect of flowcell and library preparation on the results of transcriptome sequencing using the Illumina Genome Analyzer [5]. This study includes 14 experiments on Ambion's human brain reference RNA and 28 experiments on Stratagene's universal human reference (UHR) RNA which is composed of total RNA isolated from 10 different cell lines including adenocarcinoma of mammary gland, hepatoblastoma of liver, adenocarcinoma of cervix, embryonal carcinoma of testis, glioblastoma of brain, melanoma, liposarcoma, histiocytic lymphoma (macrophase, histocyte), lymphoblastic leukemia and plasmacytoma (myeloma, B lymphocyte) (SRA Project ID: SRP001847). 2) 12 RNA-seq experiments from a comparative study on sex-specific and lineage-specific alternative splicing [16]. This study includes 6 experiments on 3 female liver samples and 6 experiments on 3 male liver samples (SRA Project ID: SRP001558). 3) 20 RNA-seq experiments from RNA-seq expression profiling study for ENCODE project common cell lines [17]. This study includes 9 RNA-seq experiments on K562 cell line produced from a female patient with chronic myelogenous leukemia (CML), 4 RNA-seq experiments on Hep2G cell line produced from a male patient with liver carcinoma, and 7 RNA-seq experiments on GM12878 cell line produced from the blood of a female donor with northern and western European ancestry by EBV transformation (SRA Project ID: SRP000228). 4) 6 RNA-seq experiments from an assessment study on technical reproducibility of RNA-seq and its comparison with gene expression arrays [1]. This study includes 3 RNA-seq experiments on liver and 3 RNA-seq experiments on kidney samples of a single human male (SRA Project ID: SRP000225). 5) 6 RNA-seq experiments from a study on human tissue alternative splicing complexity [18]. This study includes 1 RNA-seq experiments on each of the brain, cerebral cortex, heart, skeletal muscle, lung and liver samples (SRA Project ID: SRP000626).

### Short Read Alignment

Sequence read files were downloaded from NCBI SRA in FASTQ format. Raw sequence reads were aligned to the human reference genome (UCSC hg18, NCBI build 36) using TopHat [19] (Version 1.0.13) that runs on Bowtie (Version 0.12.7). Only unique alignments to the reference were considered.

### Gene counts

R/Bioconductor package Rsamtools was used to read sequence alignment results in SAM/BAM format. R/Bioconductor package GenomicRanges was used to download NCBI RefSeq gene annotations and to count total number of sequence reads on each annotated region, *gene counts*. A large matrix of gene counts (number of transcripts by number of experiments, 28,005 by 86) was constructed and saved as a simple text file.

### DFI calculation

Differential Feature Index (DFI) for a specific gene is defined as the variation of average pair-wise ratios between this gene and all the other genes across multiple samples. Out of n genes in total, for every combination of two genes *j *and *k *and in experiment *i*, log2-transformed ratios of gene counts *g_ji _*and *g_ki _*are calculated. Array *G_jk _*of *m *elements consists of these ratios across *m *experiments:

(2) $$\begin{array}{ccc} G_{jk} & =\left\{log_{2}\left(\frac{g_{j1}}{g_{k1}}\right),log_{2}\left(\frac{g_{j2}}{g_{k2}}\right),\cdots \;,log_{2}\left(\frac{g_{jm}}{g_{km}}\right)\right\} & \\ & ={\left\{log_{2}\left(\frac{g_{ji}}{g_{ki}}\right)\right\}}_{i=1\to m} \end{array}$$

Pair-wise variation is calculated as the standard deviation of the *G_jk _*elements:

(3) $$S_{jk}=std\left(G_{jk}\right)$$

DFI_j _for a gene *j *is calculated as the mean of all pair-wise variations, *S_jk_*:

(4) $$DFI_{j}=\frac{\sum_{k=1}^{n}S_{jk}}{n-1}$$

The code for DFI calculation is developed in Matlab. In order to avoid infinite values in log calculations, genes with 0 counts are replaced with 1. Runtime for the algorithm is O(n^2^m) where n is the number of genes and m is the number of samples.

### Other differential expression methods

R/Bioconductor packages EdgeR [9] and DESeq [10], and R t.test function were used to calculate differentially expressed genes between 2 samples of MAQC dataset. Same gene counts table as in DFI calculations were employed for EdgeR and DEseq methods, while normalized counts (RPKM) were employed for t-test function. Cuffdiff function of Cufflinks (Version 1.0.3) [12] was used to identify differentially expressed genes between 2 samples. Alignment results for 42 experiments of MAQC dataset were directly given as an input to the Cufflinks software. FDR adjusted p-values and fold changes reported by these methods were used in all of the calculations.

### qRT-PCR data

The quantitative real-time polymerase chain reaction (qRT-PCR) data on Ambion's human brain reference RNA and Stratagene's UHR RNA samples were downloaded from Gene Expression Omnibus (GEO), GSE5350 Series, 4 Brain and 4 UHR Taqman assays [13]. Out of 997 genes in TaqMan assay, 976 were common to NCBI RefSeq gene annotations. Then, 41 genes with no expression in any of the samples were eliminated. Expression of the remaining 935 genes were considered as gold standard to evaluate accuracy of DFI ranking for RNA-seq experiments (SRA Project SRP001558) on the same samples.

## Competing interests

The authors declare that they have no competing interests.

## Authors' contributions

H.G.O. developed, implemented and evaluated DFI method with K.H.. H.G.O. wrote the manuscript. J.D.P. and K.H. directed the project and edited the manuscript.

## Supplementary Material

Additional file 1 **Selected gene features and IPA functional annotations for liver and brain samples**. Click here for file

Additional file 2 **Selected gene features and IPA functional annotations for liver carcinoma, blood and leukaemia samples from ENCODE project**. Click here for file

Additional file 3 **Supplementary figures S1 to S4**. Click here for file
