# Supplementary material for: DFI: gene feature discovery in RNA-seq experiments from multiple sources
Source: BMC Genomics. 2012 Dec 17;13(Suppl 8):S11. doi: 10.1186/1471-2164-13-S8-S11 (PMC3535702; doi:10.1186/1471-2164-13-S8-S11)

Supplementary Figures

Supplementary Figure 1. Number of sequence reads that are aligned and not aligned to the reference genome for the 86 RNA-seq experiments.

Supplementary Figure 2. Scatter plots of DFI values, EdgeR p-values and DESeq p-values for all gene features (x-axis) versus genes with low (top, a,b,c) and high (bottom,d,e,f) counts excluded (y-axis).

Supplementary Figure 3. ROC curve (mean +/- standard deviation) for 100 random MAQC subsets. We randomly selected 3 samples from each of the Brain and UHR samples of MAQC dataset and calculated DFI values for the subset. Mean and range of standard deviation for 100 such calculations were shown in the figure.

Supplementary Figure 4. Scatter plot showing DFI values calculated for 86 RNA-seq experiments (x-axis) and log10 of the gene lengths.

Supplementary Figure 1

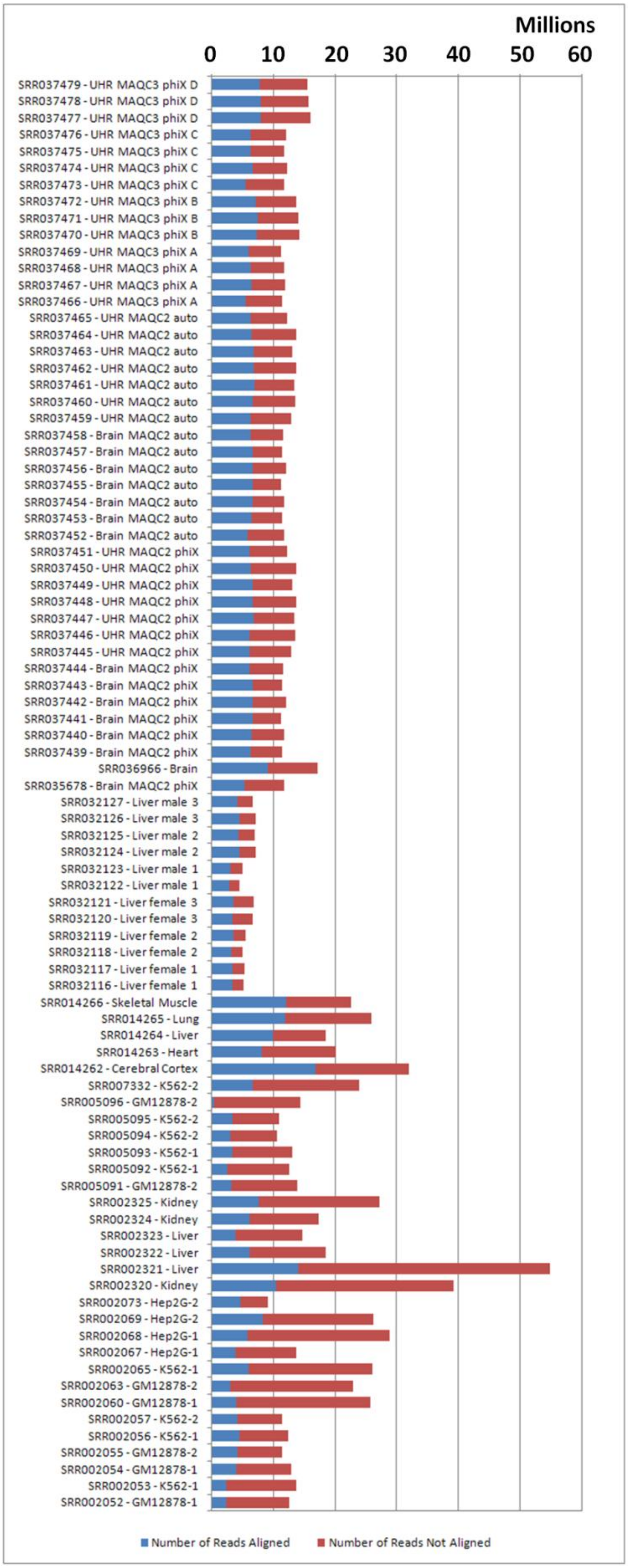

Supplementary Figure 2

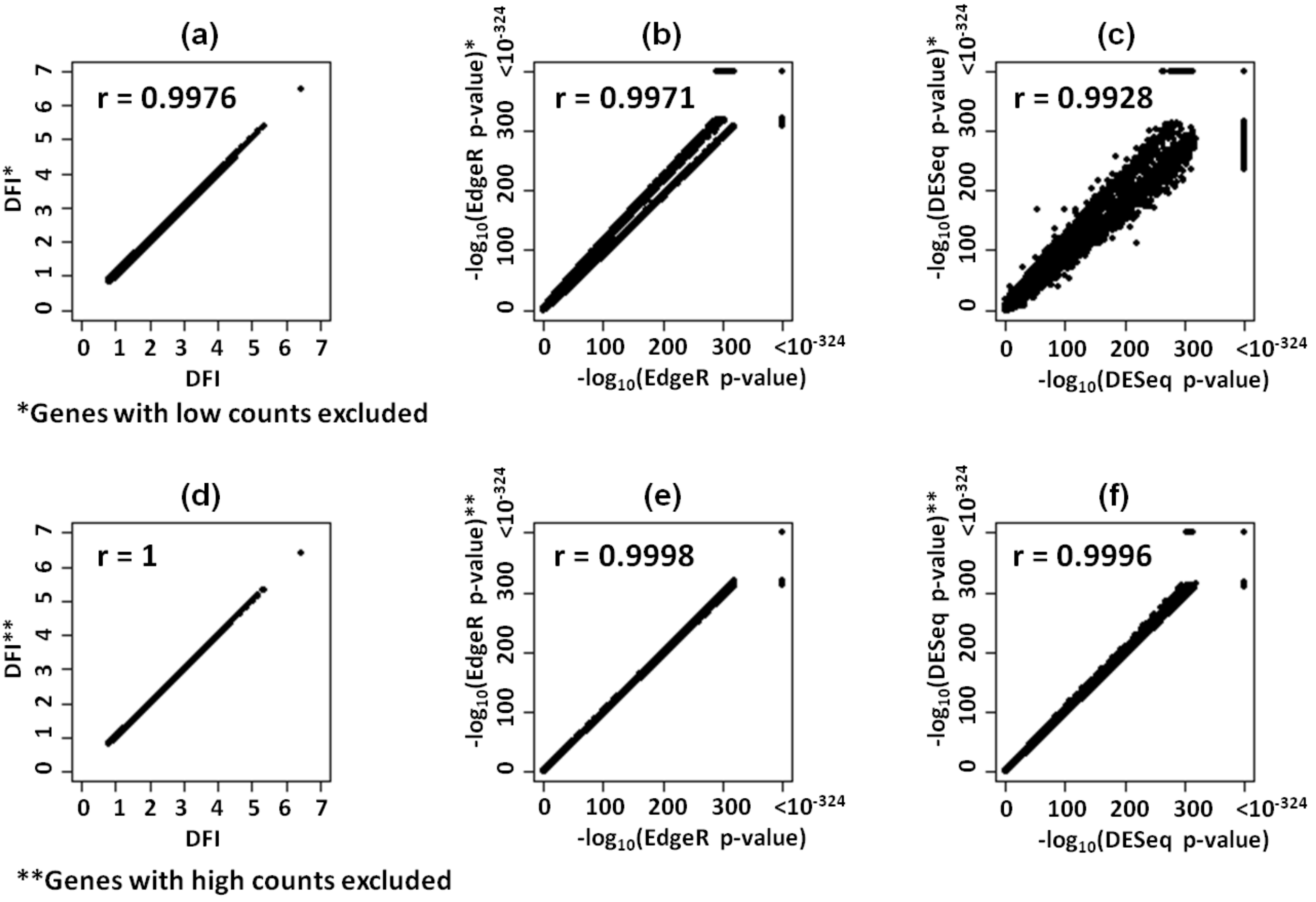

Supplementary Figure 3

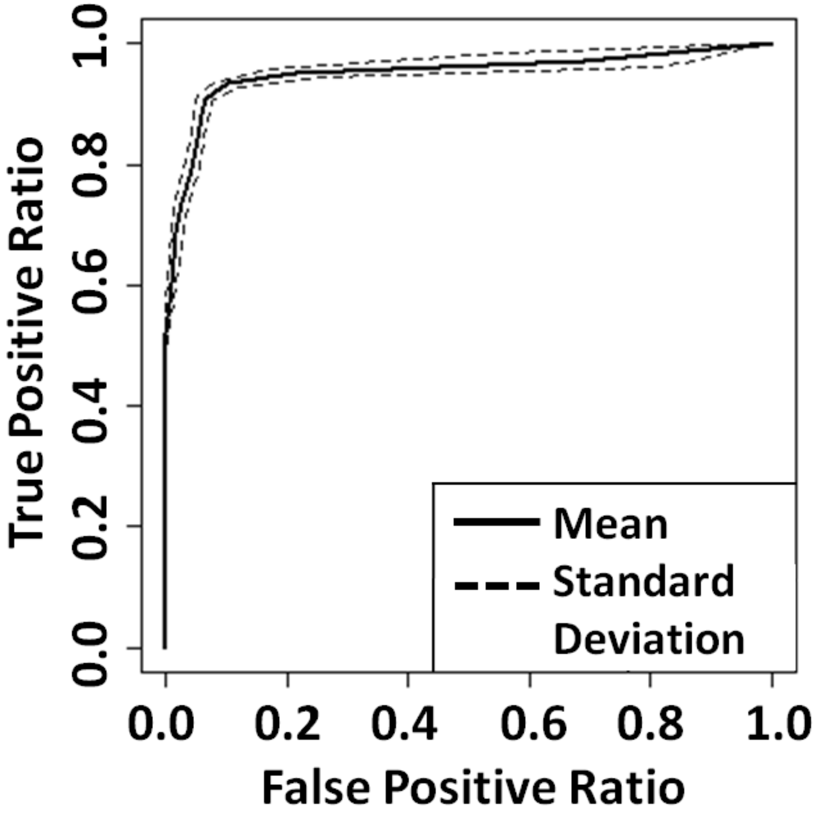

Supplementary Figure 4

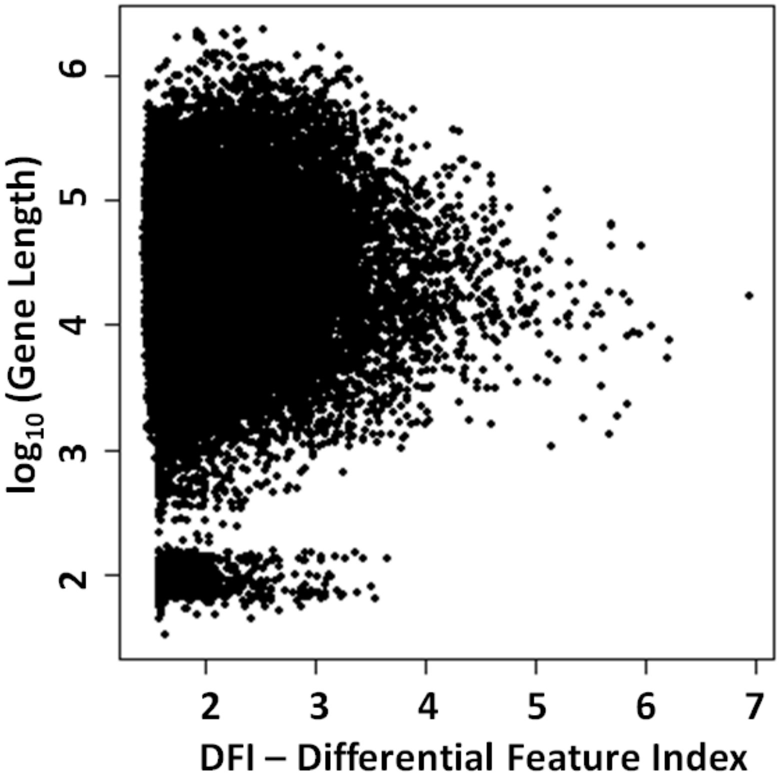

Supplement: Additional file 3 — Supplementary figures S1 to S4. [file 1471-2164-13-S8-S11-S3.pdf]
